# “The association of cardiometabolic diseases and related medications with cognitive performance: a cross-sectional observational study from Central Poland”

**DOI:** 10.1186/s12877-025-06322-9

**Published:** 2025-08-16

**Authors:** Bartłomiej K. Sołtysik, Anna Cieślak-Skubel, Agnieszka Gutowska, Elisio Costa, Tomasz Kostka

**Affiliations:** 1 Department of Geriatrics, Healthy Ageing Research Centre (HARC), Medical University of Lodz, Haller Sqr. No. 1, Lodz, 90-647 Poland; 2 Laboratory of Biochemistry, Department of Biological Sciences, Faculty of Pharmacy and Porto4Ageing EIP-AHA Reference Site, UCIBIO-REQUIMTE, University of Porto, Rua de Jorge Viterbo Ferreira, 228, Porto, 4050-313 Portugal

**Keywords:** Dementia, Cognitive decline, Cardiovascular disease, Cardiometabolic medications

## Abstract

**Introduction:**

Global ageing, the rising prevalence of cardiometabolic diseases and dementia becomes increasingly important public health topic. This relation has evoked extensive research efforts aimed at finding the link between cardiovascular health and cognitive decline. Cardiometabolic medications, widely prescribed for their beneficial effects on vascular health, are being closely examined for their potential influence on cognitive health and their capacity to modify the risk of dementia.

**Methods:**

In this cross-sectional study, 1735 individuals from Central Poland were investigated on cardiometabolic diseases, treatments, and cognitive function. Cognitive performance was evaluated using the Mini-Mental State Examination (MMSE) dichotomized to ≥ 24 points and < 24 points.

**Results:**

The logistic regression revealed a strong association between age (OR:1.07, 95% CI 1.05–1.08, *p* < 0.001) and stroke (OR:1.43, 95% CI 1.30–1.84, *p* < 0.001) with worse MMSE dichotomized score. Similarly, insulin usage was linked to an increased risk of low MMSE (OR:1.33, 95%CI: 1.06–1.73, *p* = 0.008). In contrast, BMI (OR:0.96, 95%CI 0.94–0.98, *p* = 0.001), lipid disorders (OR:0.69, 95% CI 0.48–0.92, *p* = 0.01), use of calcium channels blockers (OR:0.71, 95% CI 0.48–0.94, *p* = 0.04), alpha-blockers (OR:0.54, 95% CI 0.23–0.86, *p* = 0.004) and angiotensin II receptor antagonists (OR 0.56, 95% CI 0.29–0.83, *p* = 0.001), were associated with normal cognitive functions (R^2^ = 0.08, *p* < 0.001).

**Conclusions:**

The results present the complex connection between cardiometabolic disorders, their treatment and cognitive functioning. The research highlights the necessity for a tailored approach in prescribing cardiovascular medications, considering their potential link with cognitive health. Especially the role of angiotensin II receptor antagonists merits further studies.

## Introduction

The challenge of dementia looms large on the global health horizon, stimulated by the ageing process [1]. The problem particularly refers to regions like Central Poland where the pace of society aging is extremely rapid [2]. Thus, intensive research into the leading age-related diseases like cardiometabolic conditions and dementia, is urgently needed. The link between cardiometabolic diseases and dementia is not only sourced in age correlation [3]. Current knowledge points out that various cardiovascular conditions—such as hypertension, hypercholesterolemia, atherosclerosis, stroke, atrial fibrillation, and heart failure—significantly increase the risk of cognitive decline [4–6]. Initially, it was believed that such effects were limited to vascular dementia (VaD); however, emerging evidence has suggested that cardiovascular risk also plays a crucial role in the development of Alzheimer’s disease (AD) [7, 8]. Common risk factors for cardiovascular disease and dementia include abnormal body mass index (BMI), nicotine addiction, lipid disorders, hypertension, diabetes, poor diet, lack of physical activity, fatty liver disease, and genetic predispositions [3]. Mechanisms potentially responsible for this relationship include neuroinflammation [9, 10], oxidative stress [11, 12], disturbances in mitochondrial metabolism [13, 14], and dysregulation of heart rate with remarkably reduced heart rate variability [15, 16]. In addition, hyperglycaemia and insulin resistance, beyond causing inflammation, may also contribute to the accumulation of beta-amyloid (Aβ) and tau protein, markers of AD [17, 18]. Yet, this area still presents contradictory or inconclusive findings.

Certain medications used in managing cardiovascular conditions are identified as agents that can alter the risk of dementia. However, the existing studies frequently narrow their focus to antihypertensive medications, often overlooking other commonly used treatments like anticoagulants, hypouricemic, lipid-lowering, and hypoglycaemic drugs, or they examine these medications in a selective way. Moreover, some findings appear to be contradicting. An example is angiotensin receptor antagonists (ARBs), with research presenting diversity from negative impacts, through neutral [19], to positive effects [20] on cognitive performance. Another study suggests that the effect of antihypertensive medications on lowering the risk of dementia is associated with substances that maintain normal cerebral blood flow, such as calcium channels blockers (CCBs), angiotensin converting enzyme inhibitors (ACEI), and ARBs. This observation may potentially explain the absence of a similar effect in the case of diuretics [21].

Statins and other lipid-lowering agents are used to control hypercholesterolemia, the condition linked to the development of dementia [22, 23]. These medications might bring neuroprotective benefits by reducing the Aβ plaques formation and revealing anti-inflammatory effects, yet the evidence supporting such a role in dementia prevention is still inconsistent. Anticoagulants, crucial for stroke prevention in patients with atrial fibrillation and other cardiovascular diseases, also demand consideration for the possible impact on dementia risk [24, 25]. By preventing cerebrovascular accidents, these medications could potentially reduce the incidence of VaD. However, the balance between the cognitive benefits and the risk of bleeding is essential. Diabetes medications treat insulin resistance and hyperglycaemia, conditions linked with the pathogenesis of AD. The role of these drugs in modulating dementia risk highlights the importance of glycaemic control in patients with cardiometabolic disorders, suggesting that interventions improving insulin sensitivity might bring cognitive benefits [26–28]. Lastly, treatment targeting metabolic syndrome components like obesity, dyslipidemia, and hypertension, indicates the connection of cardiovascular and cognitive health [29, 30].

As summarized above, the existing knowledge of the overlapping features of cardiometabolic conditions and dementia is still insufficient, especially in adults of advanced age. This paper aims to explore the connection between cardiovascular diseases, their pharmacological management, and cognitive functioning, with an emphasis on the older population in Central Poland. To our knowledge, this is the first study in this demographic region that simultaneously evaluates a broad range of both cardiometabolic diseases and medication groups in relation to cognitive performance. The inclusion of real-world, hospitalized patients further enhances the clinical relevance and generalizability of our findings. By including the wide spectrum of cardiometabolic diseases and associated medications, our study seeks to provide an understanding on how these factors may act in the context of cognitive decline.

## Materials and methods

### Study design

The chosen study sample comprised individuals who were hospitalized in the Geriatric Department at the Central Veterans Hospital in Lodz, Poland. Recruitment took place from January 2012 to September 2023. During the period 2020–2022, the department worked periodically as a COVID-19 ward. Selection criteria included admission to the department, age 60 and above, effective communication ability, availability of complete data, and informed consent. Out of the 4600 hospitalized older patients, 1965 patients were excluded (readmission hospitalizations, severe dementia or terminal illness). Further 900 patients were excluded due to lack of MMSE or BMI. After data analysing, 1735 patients (1233 women and 502 men) were qualified to the study. The testing group is representative of clinically vulnerable elderly populations. While the hospital-based setting may limit generalizability to the broader community, the cohort reflects typical European patterns of aging, multimorbidity, and medication use, enhancing the study’s relevance to real-world clinical scenarios.

### Cognition assessment

The evaluation of cognitive function was conducted through the application of the Mini Mental State Examination, a simple tool employed for assessing the cognitive function [31]. The assessment contains various tasks, including orientation in time and place, memory, attention and counting, recall, language function, repetition, following commands, and constructional praxis. Obtaining a maximum score of 30 points in the test is possible. Scores ranging from 27 to 30 suggest normal cognitive function, while scores between 24 and 26 indicate mild cognitive impairment (MCI). Scores with the range of 19 to 23 signify mild dementia, 11 to 18 indicate moderate dementia, and 0 to 10 suggest severe dementia. MMSE data were used as continuous variable and dichotomized to ≥ 24 points and < 24 points, indicative for a diagnosis of dementia.

### Concomitant diseases

Various health conditions were examined, including arterial hypertension, diabetes, lipid disorders, stroke, coronary artery disease, myocardial infarction, atrial fibrillation, heart failure, Chronic Kidney Disease (CKD). CKD was determined by glomerular filtration rate (GFR) below 60 mL/min/1.73 m2 using the BIS1 formula [32]. The information about cardiometabolic medications in division by main groups was reported, and polypharmacy was defined as five or more medications taken daily.

### Statistical analysis

The Shapiro–Wilk test was used to assess the normality of distribution. Given that several variables did not exhibit a normal distribution, data were presented as both the mean ± standard deviation (SD) and median (quartiles). Due to non-normal distribution and variance heterogeneity, the Mann–Whitney U-test was employed to compare quantitative variables. Qualitative variables were evaluated using a Chi-square test. Spearman correlation coefficients were calculated to determine relationships between quantitative variables. To indicate the disorders associated with the cognitive decline in our study, a two-step approach was adopted. Initially, the Mann Whitney test was applied to compare chronic diseases linked with significantly higher or lower MMSE. Subsequently, multiple logistic regression with age, BMI, sex, and all the diseases and medications groups was employed to indicate which variables are associated with worse (< 24 points) MMSE. Finally, the results were verified in stepwise forward regression. The limit for statistical significance was set at *p* ≤ 0.05. Correction for multiple testing was not applied, as the analyses were based on a limited set of a priori, theory-driven hypotheses rather than data-driven exploration, thereby reducing the likelihood of overstated Type I error. All statistical analyses were carried out using Statistica 13.1.

 Ethical approval for this study was obtained in accordance with the Declaration of Helsinki guidelines and was granted by the Ethics Committee of the Medical University of Lodz (Approval Number: RNN/67/23/KE). Patients provided informed consent for all diagnostic and therapeutic procedures during hospitalization, and confidentiality was maintained for all gathered data.

## Results

Table 1 displays the primary distinctions between females and males. Women were older, with worse MMSE, and exhibited a higher prevalence of arterial hypertension, lipid disorders, heart failure, and CKD. Men were more frequently diagnosed with myocardial infarction. Regarding medication, females were significantly more likely to use beta-blockers, CCBs, ARBs, and diuretics. In contrast, males were more frequently prescribed thienopyridine antiplatelets, vitamin K antagonists, alpha-blockers, and insulin.

**Table 1:** Comparison of women and men in terms of basic anthropometric data, diseases and medication

	Women *n* = 1233(71.06%)	Men *n* = 502(28.94%)	*p*-value
Variable			
Age; mean ± SD (median and quartiles)	81.99 ± 7.64 83(77–88)	80.84 ± 8.09 82(7–87)	*p* = 0.005^(U)^
Body mass, kg; mean ± SD (median and quartiles)	65.24 ± 15.25 63(55–74)	75.47 ± 15.06 73 (65–84)	*p* < 0.001^(U)^
BMI, m/kg²; mean ± SD (median and quartiles)	26.76 ± 5.71 26.03 (22.83–30.08)	26.34 ± 5.00 25.78(23.05–29.05)	*p* = 0.31^(U)^
MMSE	22.27 ± 6.46 24 (19–27)	22.59 ± 7.07 25 (19–28)	*p* = 0.03^(U)^
Disease			
Arterial hypertension; n (%)	1049 (85.08%)	389 (77.49%)	*p* < 0.001^(chi2)^
Diabetes mellitus; n (%)	375 (30.44%)	174 (34.66%)	*p* = 0.08^(chi2)^
Lipid disorders; n (%)	597 (48.42%)	188 (37.45%)	*p* = 0.001^(chi2)^
Stroke; n (%)	215 (17.44%)	85 (16.93%)	*p* = 0.802^(chi2)^
Coronary artery disease; n (%)	407 (33.01%)	182 (36.25%)	*p* = 0.19^(chi2)^
Myocardial infarction; n (%)	105 (8.52%)	80 (15.94%)	*p* < 0.001^(chi2)^
Atrial fibrillation; n (%)	278 (22.55%)	2127 (25.30%)	*p* = 0.21^(chi2)^
Heart failure; n (%)	692 (56.12%)	253 (50.40%)	*p* = 0.02^(chi2)^
Chronic kidney disease; n (%)	592 (48.01%)	212 (42.23%)	*p* = 0.02^(chi2)^
Medications			
NOAC; n (%)	150 (8.13%)	83 (10.55%)	*p* = 0.44^(chi2)^
Acetylsalicylic acid; n (%)	432 (35.04%)	176 (35.06%)	*p* = 0.799^(chi2)^
Thienopyridine antiplatelets; n (%)	46 (3.732%)	31 (6.18%)	*p* = 0.02^(chi2)^
Vitamin K antagonists; n (%)	42 (3.41%)	30 (5.98%)	*p* = 0.01^(chi2)^
Beta-blockers; n (%)	754 (61.207%)	270 (53.89%)	*p* = 0.001^(chi2)^
CCBs; n (%)	382 (30.98%)	121 (24.10%)	*p* = 0.004^(chi2)^
Alpha-blockers; n (%)	26 (2.11%)	183 (36.45%)	*p* < 0.001^(chi2)^
ACEI; n (%)	470 (38.12%)	178 (35.46%)	*p* = 0.29^(chi2)^
ARBs; n (%)	252 (20.44%)	74 (14.74%)	*p* = 0.005^(chi2)^
Diuretics; n (%)	688 (55.80%)	249 (49.70%)	*p* = 0.02^(chi2)^
Statins; n (%)	568 (46.14%)	251 (50.00%)	*p* = 0.14^(chi2)^
Allopurinol; n (%)	142 (11.53%)	62 (12.35%)	*p* = 0.62^(chi2)^
Oral antidiabetic agents; n (%)	247 (20.03%)	121 (24.105%)	*p* = 0.060^(chi2)^
Insulin; n (%)	82 (6.65%)	48 (9.56%)	*p* = 0.036^(chi2)^
Polypharmacy (at least 5 medications)	731 (59.24%)	287 (57.29%)	*p* = 0.45^(chi2)^

*BMI* body mass index,*MMSE* mini mental state examination,*NOAC* non-vitamin K antagonist oral anticoagulants, * CCBs* calcium channel blockers, *ACEI* angiotensin converting enzyme inhibitors, * ARBs* angiotensin II receptor blockers

Chi^2^- chi^2^ test

U- Mann-Whitey test

In the next step (Table 2) we calculated the MMSE score comparison based on sex, specific diseases, or medication usage. Women with arterial hypertension, lipid disorders, and those taking CCBs, ARBs and oral antidiabetic agents demonstrated higher MMSE scores. Conversely, females with a history of stroke, and chronic heart failure, exhibited significantly lower MMSE scores. In the male population, individuals with lipid disorders and those taking ARBs showed significantly higher MMSE results. Conversely, male subjects with a previous stroke and insulin usage presented significantly lower MMSE scores. Additionally, correlations for age and BMI versus MMSE were calculated. In the whole studied population, MMSE expressed significantly negative correlation with age (rho = − 0.29, *p* < 0.001), and positive with BMI (rho = 0.171, *p* < 0.001). Similar correlations were observed separately in women and men (women: rho = − 0.321, *p* < 0.001; rho = 0.214, *p* < 0.001; men rho = − 0.223, *p* < 0.001; rho = 0.086, *p* = 0.05; respectively).

**Table 2:** Comparison of values of MMSE between subjects with and without particular disease or medication in division by sex

Disease	Sex	MMSE				p value
		In patients with presence of particular disease		In patients without particular disease		
		Mean ± SD	Median (quartiles)	Mean ± SD	Median (quartiles)	
Arterial hypertension	Women	22.48 ± 6.32	24 (19-27)	21.02 ± 7.09	22 (17-27)	p = 0.01^(U)^
	Men	22.67 ± 7.02	25 (19-28)	22.32 ± 7.29	24 (17-28)	p = 0.24^(U)^
Diabetes mellitus	Women	22.53 ± 6.79	24 (19-28)	22.15 ± 6.32	24 (18-27)	p = 0.09^(U)^
	Men	21.85 ± 7.23	23 (18-28)	22.98 ± 6.97	25 (20-28)	p = 0.06^(U)^
Lipid disorders	Women	23.36 ± 6.05	25 (20-28)	21.24 ± 6.67	22 (17-27)	p<0.001^(U)^
	Men	23.68 ± 6.82	26 (21-29)	21.93 ± 7.15	24 (18-28)	p<0.001^(U)^
Previous stroke	Women	20.71 ± 6.51	22 (17-25)	22.60 ± 6.41	24 (19-28)	p<0.001^(U)^
	Men	18.38 ± 9.74	22 (13-26)	22.99 ± 6.82	25 (19-28)	p = 0.004^(U)^
Coronary artery disease	Women	22.44 ± 6.15	24 (19-27)	22.18 ± 6.62	24 (18-28)	p = 0.89^(U)^
	Men	22.42 ± 7.28	24 (19-28)	22.69 ± 6.96	25 (19-28)	p = 0.69^(U)^
Previous myocardial infarction	Women	21.77 ± 6.55	24 (19-27)	22.31 ± 6.46	24 (19-27)	p = 0.35^(U)^
	Men	22.85 ± 6.65	24 (19-28)	22.54 ± 7.15	25 (19-28)	p = 0.98^(U)^
Atrial fibrillation	Women	22.16 ± 5.87	23 (19-27)	22.30 ± 6.63	24 (18-28)	p = 0.21^(U)^
	Men	22.13 ± 7.34	24 (18-28)	22.74 ± 6.98	25 (19-28)	p = 0.45^(U)^
Heart failure	Women	21.75 ± 6.30	23 (18-27)	22.93 ± 6.61	24 (18-28)	p<0.001^(U)^
	Men	22.23 ± 6.98	24 (19-28)	22.95 ± 7.16	26 (20-28)	p = 0.10^(U)^
Chronic kidney disease	Women	22.02 ± 6.34	23 (19-27)	22.49 ± 6.57	24 (18-28)	p = 0.059^(U)^
	Men	22.29 ± 6.77	24 (19-28)	22.73 ± 7.29	25 (19-28)	p = 0.24^(U)^

Medications	Sex	In patients with particular drug intake		In patients without particular drug intake		p value
		Mean ± SD	Median (quartiles)	Mean ± SD	Median (quartiles)	
NOAC	Women	22.50+6.08	24 (18-27)	22.23+6.53	24 (19-27)	p = 0.81^(U)^
	Men	22.58 ± 7.35	25 (19-28)	22.59+7.03	25 (19-28)	p = 0.91^(U)^
Acetylsalicylic acid	Women	22.63 ± 6.34	24 (19-27)	22.07+6.53	23 (18-27)	p = 0.11^(U)^
	Men	23.25 ± 6.51	25 (21-28)	22.23 ± 7.34	24 (18-28)	p = 0.27^(U)^
Thienopyridine antiplatelets	Women	22.94 ± 6.79	25 (20-28)	22.24 ± 6.45	24 (19-27)	p = 0.30^(U)^
	Men	23.45 ± 7.30	27 (20-29)	22.53 ± 7.06	25 (19-28)	p = 0.33^(U)^
Vitamin K antagonists	Women	22.57 ± 6.36	24 (18-27)	22.26 ± 6.47	24 (19-27)	p = 0.80^(U)^
	Men	23.23 ± 6.45	24 (21-28)	22.55 ± 7.11	25 (19-28)	p = 0.73^(U)^
Beta-blockers	Women	22.51 ± 6.29	24 (19-27)	21.90 ± 6.72	23 (18-28)	p = 0.18^(U)^
	Men	22.19 ± 7.21	24 (18-28)	23.10 ± 6.86	26 (20-28)	p = 0.11^(U)^
CCBs	Women	23.39 ± 5.49	25 (20-28)	21.76 ± 6.80	23 (18-27)	p<0.001^(U)^
	Men	22.97 ± 7.07	25 (20-29)	22.47 ± 7.08	25 (18-28)	p = 0.44^(U)^
Alpha-blockers	Women	24.00 ± 4.18	25 (20-27)	22.23 ± 6.50	24 (18-27)	p = 0.33^(U)^
	Men	23.22 ± 6.57	25 (21-28)	22.23 ± 7.33	24 (18-28)	p = 0.20^(U)^
ACEI	Women	22.34 ± 6.23	24 (19-27)	22.22 ± 6.61	24 (18-28)	p = 0.92^(U)^
	Men	22.84 ± 6.83	25 (20-28)	22.45 ± 7.21	24 (18-28)	p = 0.71^(U)^
ARBs	Women	23.89 ± 5.71	25 (21-29)	21.85 ± 6.58	23 (18-27)	p<0.001^(U)^
	Men	24.22 ± 6.29	26 (21-29)	22.31 ± 7.17	24 (18-28)	p = 0.01^(U)^
Diuretics	Women	22.68 ± 5.94	24 (19-27)	21.75 ± 7.04	24 (18-28)	p = 0.13^(U)^
	Men	22.78 ± 6.78	24 (19-28)	22.43 ± 7.36	25 (18-28)	p = 0.92^(U)^
Statins	Women	22.00 ± 6.76	23 (18-28)	22.57 ± 6.10	24 (19-27)	p = 0.29^(U)^
	Men	23.11 ± 6.35	25 (20-28)	22.07 ± 7.71	25 (18-28)	p = 0.39^(U)^
Allopurinol	Women	23.21 ± 5.31	25 (20-27)	22.14 ± 6.59	24 (18-27)	p = 0.19^(U)^
	Men	23.61 ± 5.84	25 (21-28)	22.45 ± 7.22	25 (18-28)	p = 0.52^(U)^
Oral antidiabetic agents	Women	23.33 ± 6.02	25 (20-28)	22.00 ± 6.55	23 (18-27)	p = 0.002^(U)^
	Men	22.71 ± 6.76	24 (18-29)	22.55 ± 7.18	25 (19-28)	p = 0.91^(U)^
Insulin	Women	22.23 ± 6.49	24 (18-27)	22.79 ± 6.06	25 (19-27)	p = 0.52^(U)^
	Men	19.18 ± 8.12	21 (15-25)	22.95 ± 6.86	25 (19-280)	p<0.001^(U)^

Medications	Sex	In patients with polypharmacy		In patients without polypharmacy		p value
		Mean ± SD	Median (quartiles)	Mean ± SD	Median (quartiles)	
Polypharmacy (at least 5 medications)	Women	22.54 ± 6.19	24 (19-27)	21.91 ± 6.83	23 (18-28	p = 0.35^(U)^
	Men	22.44 ± 6.94	24 (19-28)	22.71 ± 7.30	25 (19-28)	p = 0.25^(U)^

*MMSE* mini mental state examination, *NOAC* non-vitamin K antagonist oral anticoagulants, * CCBs* calcium channel blockers, * ACEI*angiotensin converting enzyme inhibitors, * ARBs* angiotensin II receptor blockers

U- Mann-Whitey test

In the subsequent phase of the study, we have analysed the age differences among participants based on their health conditions and medication usage (Table 3). Women with conditions such as hypertension, coronary artery disease, previous myocardial infarction, atrial fibrillation, heart failure, and CKD were notably older. Conversely, women with lipid disorders were younger than those without such condition. Women taking beta-blockers and diuretics were significantly older, while those on insulin and oral antidiabetic therapy were younger. For men, those with coronary artery disease, atrial fibrillation, heart failure, and chronic kidney disease were older than their counterparts without these conditions. The intake of beta-blockers, alpha-blockers, and diuretics was associated with older age, whereas insulin therapy (borderline significance) was linked to a younger age.

**Table 3:** Comparison of age between subjects with and without particular disease or medication in division by sex

Disease	Sex	Age				p value
		In patients with presence of particular disease		In patients without particular disease		
		Mean ± SD	Median (quartiles)	Mean ± SD	Median (quartiles)	
Arterial hypertension	Women	82.32 ± 7.48	83 (78–88)	80.05 ± 8.25	81 (74–86)	p = 0.0001
	Men	81.19 ± 7.76	82 (76–87)	79.64 ± 9.06	81 (73–87)	p = 0.12
Diabetes mellitus	Women	81.58 ± 7.32	82 (77–87)	82.17 ± 7.78	83 (77–88)	p = 0.07
	Men	80.10 ± 7.08	80 (75–86)	81.24 ± 8.55	82 (75–88)	p = 0.08
Lipid disorders	Women	80.44 ± 7.50	81 (75–86)	83.43 ± 7.49	85 (80–89)	p < 0.001
	Men	79.34 ± 8.28	80 (73–86)	81.75 ± 7.84	83 (76–88)	p = 0.001
Previous stroke	Women	82.67 ± 7.64	84 (78–88)	81.84 ± 7.64	83 (77–87)	p = 0.06
	Men	80.70 ± 7.71	82 (75–86)	80.87 ± 8.17	82 (75–87)	p = 0.83
Coronary artery disease	Women	83.48 ± 6.77	85 (79–88)	81.25 ± 7.99	83 (77–88)	p < 0.001
	Men	82.47 ± 7.81	84 (77–88)	79.92 ± 8.10	81 (74–86)	p < 0.001
Previous myocardial infarction	Women	83.76 ± 6.95	85 (80–88)	81.82 ± 7.69	84 (77–88)	p = 0.008
	Men	81.73 ± 7.66	82 (76–87)	80.68 ± 8.16	82 (75–87)	p = 0.30
Atrial fibrillation	Women	83.71 ± 6.87	85 (80–89)	81.48 ± 7.78	82 (77–87)	p < 0.001
	Men	83.29 ± 7.19	84 (78–89)	80.01 ± 8.21	81 (74–86)	p < 0.001
Heart failure	Women	83.51 ± 7.17	85 (79–89)	80.04 ± 7.80	81 (74–86)	p < 0.001
	Men	82.73 ± 7.47	84 (78–88)	78.93 ± 8.25	79 (73–85)	p < 0.001
Chronic kidney disease	Women	84.26 ± 6.55	85 (80–89)	79.88 ± 7.97	81 (74–86)	p < 0.001
	Men	83.67 ± 7.05	84 (78–89)	78.78+8.18	79 (72–85)	p < 0.001

Medications	Sex	In patients with particular drug intake		In patients without particular drug intake		p value
		Mean ± SD	Median (quartiles)	Mean ± SD	Median (quartiles)	
NOAC	Women	81.44 ± 7.67	83 (75–88)	82.08 ± 7.64	83 (77–88)	p = 0.37
	Men	81.56 ± 7.99	82 (75–88)	80.71 ± 8.10	82 (75–87)	p = 0.34
Acetylsalicylic acid	Women	82.50 ± 7.10	83 (78–88)	81.71 ± 7.91	83 (76–88)	p = 0.14
	Men	81.18 ± 7.85	82 (76–87)	80.66 ± 8.21	82 (75–87)	p = 0.51
Thienopyridine antiplatelets	Women	83.93 ± 7.20	85 (80–89)	81.91 ± 7.65	83 (77–88)	p = 0.06
	Men	81.25 ± 9.59	85 (73–89)	80.82 ± 7.99	82 (75–87)	p = 0.47
Vitamin K antagonists	Women	80.80 ± 8.18	84 (76–86)	82.02 ± 7.62	83 (77–88)	p = 0.49
	Men	81.30 ± 6.95	82 (77–87)	80.81 ± 8.16	82 (75–87)	p = 0.74
Beta-blockers	Women	82.63 ± 7.29	84 (78–88)	80.96 ± 8.08	82 (75–87)	p < 0.001
	Men	81.78 ± 7.92	83 (76–88)	79.78 ± 8.16	81 (74–86)	p < 0.001
CCBs	Women	82.57 ± 6.84	83 (79–87)	81.72 ± 7.97	83 (76–88)	p = 0.16
	Men	80.42 ± 7.66	81 (75–86)	80.98 ± 8.22	82 (75–87)	p = 0.53
Alpha-blockers	Women	81.53 ± 9.42	82 (75–88)	82.00 ± 7.61	83 (77–88)	p = 0.63
	Men	82.29 ± 6.47	83 (78–87)	80.01 ± 8.78	81 (73–87)	p = 0.006
ACEI	Women	82.25 ± 7.21	83 (78–87)	82.23 ± 7.63	83 (78–88)	p = 0.49
	Men	81.17 ± 8.04	82 (75–87)	80.66 ± 8.12	82 (75–87)	p = 0.57
ARBs	Women	81.51 ± 7.42	83 (77–87)	82.11 ± 7.70	83 (77–88)	p = 0.21
	Men	79.81 ± 7.56	80 (75–86)	81.02 ± 8.17	82 (75–87)	p = 0.21
Diuretics	Women	82.89 ± 7.37	84 (79–88)	80.84 ± 7.83	82 (76–87)	p < 0.001
	Men	81.70 ± 8.01	83 (76–87)	80.02 ± 8.09	81 (74–86)	p = 0.02
Statins	Women	82.13 ± 7.30	83 (78–87)	81.83 ± 7.91	83 (77–88)	p = 0.71
	Men	81.24 ± 7.81	82 (76–87)	80.45 ± 8.35	81 (74–87)	p = 0.24
Allopurinol	Women	82.13 ± 7.54	82 (77–88)	81.97 ± 7.66	83 (77–88)	p = 0.86
	Men	81.20 ± 7.40	81 (76–86)	80.79 ± 8.18	82 (75–87)	p = 0.93
Oral antidiabetic agents	Women	81.34 ± 6.94	82 (77–86)	82.15 ± 7.80	83 (77–88)	p = 0.05
	Men	80.43 ± 7.34	81 (75–86)	80.89 ± 8.31	82 (75–87)	p = 0.38
Insulin	Women	79.85 ± 7.22	80 (75–84)	82.14 ± 7.65	83 (77–88)	p = 0.001
	Men	79.08 ± 6.34	79 (75–83)	81.03 ± 8.23	82 (75–87)	p = 0.06

*SD* standard deviation, compared by U Mann Whitney test

**Fig. 1:**
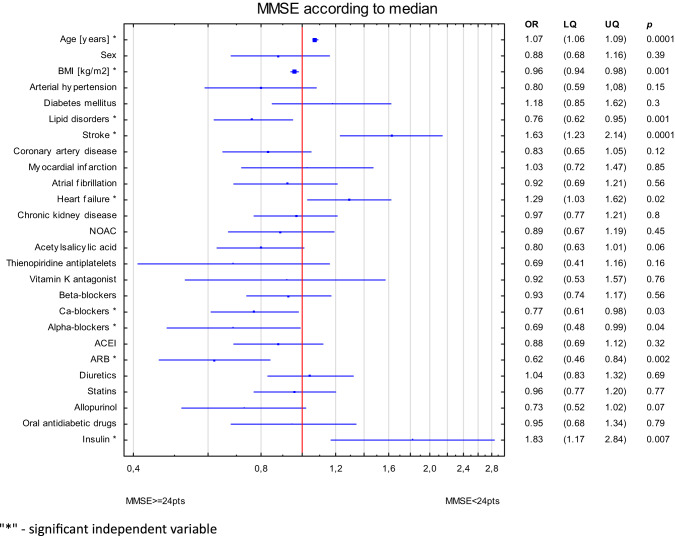
Forest plot for the MMSE divided according to median and all tested variables. “*” - significant independent variable

Consequently, variables were employed to construct a logistic regression model. The dependent variable was the MMSE dichotomized based on the median (≥ 24 points vs. <24 points. The model of multiple logistic regression was developed for all independent variables (age, BMI, sex, and all the diseases and medications groups) to emphasize in the forest plot the potential direction of the associations (Fig. 1). Advanced age, history of stroke, heart failure and insulin treatment were associated with low MMSE. In contrast, higher BMI, lipid disorders, and ARB, Ca-blockers and alpha-blockers treatment were related to high MMSE.

As a last phase of analysis, the stepwise regression was calculated to confirm the independent associations of independent variables to low MMSE. Variables such as age (OR:1.07, 95% CI1.05–1.08; *p* < 0.001), BMI (OR:0.96, 95% CI 0.94–0.98, *p* = 0.001), lipid disorders (OR:0.69, 95% CI 0.48–0.92, *p* = 0.01), stroke (OR:1.43, 95% CI 1.30–1.84, *p* < 0.001) use of CCBs (OR:0.71, 95% CI 0.48–0.94, *p* = 0.04), alpha-blockers (OR:0.54, 95% CI 0.23–0.86, *p* = 0.004), ARBs (OR 0.56, 95% CI 0.29–0.83, *p* = 0.001), insulin (OR:1.33, 95% CI 1.06–1.73, *p* = 0.008) were associated with MMSE dichotomized score (R^2^ = 0.08, *p* < 0.001).

## Discussion

The study aims to explore the association between cardiometabolic diseases, their corresponding medications, and cognitive function in older patients. Our findings indicate that health conditions and prescribed drugs appear to be linked to cognition, with associations observed in both higher and diminished cognitive functioning.

In our study, logistic regression analysis reveals that lipid disorders and BMI are linked to normal MMSE. Conversely, age and previous stroke are associated with the symptoms of dementia. Relation of heart failure was of borderline significance. Regarding pharmacotherapy, the independent association with higher MMSE values is observed with the use of CCBs, alpha-blockers and ARBs. However, insulin intake demonstrates a connection with cognitive decline.

The cognitive function assessed through the prism of cardiometabolic disorders seems intertwined. BMI correlates positively with MMSE value, indicating that higher BMI is associated with normal cognitive function. Cao et al. indicates that BMI is independent predictors of dementia, but the study concerned people aged 37–73 [33]. The relationship between BMI, age and cognitive function appears to be age-dependent. Higher BMI is associated with symptoms of dementia in middle age, while among seniors, higher BMI is linked with better cognition and decreased mortality [34]. Finally, the study in the group of two million people indicates that low body mass in middle age and old age increases the risk of dementia, whereas obesity lowers it by 29% [35]. Hypertension and past strokes are attributed to VaD [36–38] Elevated blood pressure leads to microcirculation disorders and neuronal damage [39]. Another disorder associated with hypertension is AD, as high blood pressure is linked with the accumulation of Aβ and TAU protein. Some researchers suggest that the pathophysiological changes in VaD and AD overlap [21, 40]. Hence, the presence of HA is proven to be strictly linked not only with VaD, but also AD, or MCI [21, 41, 42]. Lipid disorders, are closely associated with dementia risk [22, 43]. In our research, we found that lipid disorders were associated with reduced risk of cognitive decline. This finding was initially unexpected and contradicting to initial beliefs. However, a more thorough examination revealed that individuals with lipid disorders were notably younger than their counterparts without such conditions. Another explanation may be associated with abovementioned protective effect of higher BMI, with lipid disorders claimed as its derivative.

The Framingham post-stroke subjects had a 2.0–2.8 times higher risk of dementia compared to healthy individuals [21]. The interaction between these conditions is not limited to the loss of neurons and memory impairment [38, 44]. The connection between ischemic stroke and cerebral dementia lies in vascular and neurodegenerative processes [45–47] like the development of neurofibrillary tangles and intraneural plaques [48]. Furthermore, the accumulated Aβ in blood vessels damages the blood-brain barrier, and impaired clearance mechanisms play critical role in the development of post-stroke dementia [37, 49, 50]. Similarmechanisms may explain the relationship between heart disease and dementia. Coronary artery disease, myocardial infarction, atrial fibrillation, valvular disease, or heart failure cause hemodynamic stress, reduced blood flow to the brain, neuroinflammation, and increased coagulation what substantially elevate the dementia risk [6–8]. Furthermore, different heart failure types correlate with varied risks of developing specific dementia types, impacting patient survival rates [51]. Besides, cardiac dysfunction-induced reduction in cerebral blood flow may intensify the pathology of VaD and AD [52]. Presented study may confirm some of the mentioned associations.

Primary risk factors like BMI or lipid disorders were associated with significantly better cognitive status. They are related to younger age and their meaning seems different in advanced-age population. Arterial hypertension may be considered as intermediate factor – both disease and risk factor for cardiometabolic diseases and dementia. Stroke and heart failure results from lifetime accumulation of risk factors, are typical conditions of advanced age and were independently associated with poorer cognitive performance. In this context our results may support the hypothesis that dementia may be strongly associated with cardiometabolic risk.

Another aspect was to investigate the relationship between cardiometabolic pharmacotherapy and cognitive performance. Interesting research highlights the link between low-dose ASA use and a lower incidence of dementia in individuals with chronic heart conditions, showing a 31% decrease in AD risk, a 69% decrease in VaD risk, and a 34% decrease in overall dementia risk after a minimum of 10 years of medication use [53]. Other studies present mixed findings, indicating that aspirin might lower AD risk in older patients with ischemic stroke [54], or finding no significant link between aspirin use and dementia risk [55, 56]. The precise mechanisms through which ASA may trigger neuroprotection, such as its anti-inflammatory and anticoagulant properties potentially reducing cerebral micro-infarcts, remain only partially understood.

The knowledge for hypotensive agents is even more complex. Several studies suggest that the impact of antihypertensive medications on lowering the risk of dementia is associated with substances that help to maintain regular cerebral blood flow, such as CCBs, ACE inhibitors, and ARBs. This observation may clarify the absence of a similar effect in the case of certain medications, including diuretics [21].The findings on the connection between CCBs and dementia are conflicting, indicating that some of CCBs may help prevent cognitive deterioration [21, 57] or showing no effect [58]. Molecularly, elevated intracellular calcium can trigger neuronal death, activation of enzymes that degrade cellular components, production of reactive oxygen species, and the initiation of apoptosis [59]. Aβ in oligomeric state, play a role in the progression of AD by creating pores in the membranes of neurons, leading to excessive calcium entry, neuronal damage, oxidative stress, neuroinflammation and ultimately apoptosis. CCBs potentially reduce this adverse effect [60]. The research about alpha-blockers indicate on substance depending positive or negative effect on dementia risk [61], pointing on tamsulosin as a substance with elevated risk of cognitive decline [62] and doxazosin carrying neuroprotective effect [63]. ARBs may also offer protective effect against dementia. The OSCAR study, which involved more than 25,000 participants aged 50 and above, showed a distinct adverse relationship between eplenosartan intake and cognitive deterioration. Additionally, the study describing comparison between the impact of an ACE inhibitor plus diuretic versus an ARB plus diuretic, proved better cognitive functions with the use of the sartan-included course [21]. ARBs, by inhibiting the angiotensin II type 1 receptor, reduce blood pressure, but also reduce inflammation, improve endothelial function, and decrease oxidative stress, all of which could indirectly impact the mechanisms involved in the onset of dementia [64]. ARBs could influence against cognitive deterioration, potentially due to their capacity to penetrate the blood-brain barrier and directly influence brain tissue [64].

The impact of statins on dementia remains disputable with conflicting evidence, with studies suggesting both neuroprotective and neurodegenerative effects from these medications [65]. Insulin plays crucial roles in the brain, including learning and memory processes, which are disturbed in AD. Brain insulin resistance emerges as a pathological hallmark of AD, leading to dysregulation in metabolism, inflammation, oxidative stress, impaired insulin signalling, mitochondrial dysfunction and synaptic dysfunction [66–68]. These conditions act as molecular links between diabetes and AD. In both disorders, constant inflammation induces insulin resistance, what next exacerbates neurodegeneration and cognitive decline. Targeting inflammation, insulin resistance and insulin’s role in the metabolism of Aβ and the phosphorylation of tau, essential in AD’s pathology present a promising direction for novel AD therapies [66, 69, 70]. Our study shows that there is both a positive and negative relationship between cardiometabolic drugs and cognitive performance. CCBs, alpha-blockers and ARBs appear to be associated with better cognitive performance. Furthermore, the association of ARBs and cognitive function may designate on that group as the strongest protective effect among the various studied drug groups. Otherwise, the relation of insulin and worse cognitive performance may highlight the significance of metabolic pathways in the development of dementia. In our study, insulin was used in younger subjects indicating on long-term accumulation of cardiometabolic risk.

An important consideration in interpreting our findings is the broader geriatric context, particularly the roles of frailty, polypharmacy, and antidepressants use. Frailty, although not directly assessed in our study, is closely linked to both cardiovascular disease and cognitive decline and may influence the observed associations [71]. Similarly, while polypharmacy was common in our cohort, it was not independently associated with worse cognitive outcomes [72], though its cumulative effects may still be clinically relevant. Lastly, the lack of data on antidepressants use—despite its known impact on cognition in older adults [73]—represents a limitation and a potential confounder that should be addressed in future research.

A key strength of our study lies in its comprehensive coverage of cardiometabolic diseases, and the extensive variety of medications tested in relation to dementia. It’s also significant to mention that our research involves a notably large group of older individuals. Nonetheless, there are several limitations to our study that need attention.

This study’s cross-sectional, retrospective and observational design prevents establishing causal relationships between cardiometabolic factors, medications, and cognitive outcomes. The sample was drawn from a single geriatric hospital in Central Poland, which may limit the generalizability of findings to broader or more diverse populations. Furthermore, by focusing primarily on cardiometabolic variables, other potential contributors to cognitive decline, such as genetic, psychosocial, or lifestyle factors may not have been fully accounted for. Additionally, the study did not account for the duration of medication use, as data were limited to current prescriptions at the time of hospitalization; this may have influenced the observed associations between pharmacotherapy and cognitive function.

To address these scarcities, future prospective studies should be larger in scale, multicentric, and conducted across varied demographic groups. That would facilitate detailed and extensive conclusions.

## Conclusions

The research underscores a complex and multi-layered link between cardiometabolic diseases, medications, and dementia. It highlights that in multimorbid age-advanced population history of stroke, chronic heart failure and insulin therapy are linked to cognitive decline. These are end-stage consequences of cardiometabolic risk or indicators of its early treatment. On the other hand, BMI and some medications, including ASA, CCBs, alpha-blockers, ARBs, may confer a protective effect on developing dementia in advanced age. These results suggest treatment options within the cardiometabolic spectrum to improve cognitive outcomes. Furthermore, the importance of the duration of exposure to harmful or protective factors in the referenced studies stimulates to a revaluation of the extent to which dementia might arise from cardiometabolic disorders, or perhaps represents one of their subtypes.

## Data Availability

The authors declare that the data supporting the findings of this study are available from the corresponding author on reasonable request.

## Abbreviations

ACEI: Angiotensin converting enzyme inhibitors
AD: Alzheimer’s disease
ARB: Angiotensin II receptor blockers (Sartans)
ASA: Acetylsalicylic acid
Aβ: Beta–amyloid
BMI: Body mass index
CCB: Calcium channel blocker
CKD: Chronic kidney disease
GFR: Glomerular filtration rate
HA: Hypertension/Arterial hypertension
MCI: Mild cognitive impairment
MMSE: Mini mental state examination
NOAC: Non–vitamin K antagonist oral anticoagulants
OR: Odds ratio
SD: Standard deviation
VaD: Vascular dementia

## References

[1] Lopez OL, Kuller LH. Epidemiology of aging and associated cognitive disorders: prevalence and incidence of alzheimer’s disease and other dementias. Handb Clin Neurol. 2019;167:139–48. 31753130 10.1016/B978-0-12-804766-8.00009-1

[2] Kopiec T, Stańczyk K, Burzyńska M. Assessment of Lodz Province residents opinions on the needs for the social activation of seniors. Acta Universitatis Lodziensis Folia Oeconomica. 2022;6(357):4–23.

[3] Nordestgaard LT, Christoffersen M, Frikke-Schmidt R. Shared risk factors between dementia and atherosclerotic cardiovascular disease. Int J Mol Sci. 2022;23(17):9777. 36077172 10.3390/ijms23179777 PMC9456552

[4] Wang X, et al. Association of cardiovascular health with life expectancy free of cardiovascular disease, diabetes, cancer, and dementia in UK adults. JAMA Intern Med. 2023;183(4):340–9. 36848126 10.1001/jamainternmed.2023.0015 PMC9972243

[5] Stakos DA, et al. The alzheimer’s disease Amyloid-Beta hypothesis in cardiovascular aging and disease: JACC focus seminar. J Am Coll Cardiol. 2020;75(8):952–67. 32130931 10.1016/j.jacc.2019.12.033 PMC7042886

[6] Justin BN, Turek M, Hakim AM. Heart disease as a risk factor for dementia. Clin Epidemiol. 2013;5:135–45. 23658499 10.2147/CLEP.S30621 PMC3641811

[7] van der Velpen IF, et al. Impaired cardiac function and cognitive brain aging. Can J Cardiol. 2017;33(12):1587–96. 28966021 10.1016/j.cjca.2017.07.008

[8] Cermakova P, et al. Heart failure and alzheimer’s disease. J Intern Med. 2015;277(4):406–25. 25041352 10.1111/joim.12287 PMC4409079

[9] Tian Z, Ji X, Liu J. Neuroinflammation in vascular cognitive impairment and dementia: current evidence, advances, and prospects. Int J Mol Sci. 2022;23(11):6224. 35682903 10.3390/ijms23116224 PMC9181710

[10] Leng F, Edison P. Neuroinflammation and microglial activation in alzheimer disease: where do we go from here? Nat Rev Neurol. 2021;17(3):157–72. 33318676 10.1038/s41582-020-00435-y

[11] Chen Z, Zhong C. Oxidative stress in alzheimer’s disease. Neurosci Bull. 2014;30(2):271–81. 24664866 10.1007/s12264-013-1423-y PMC5562667

[12] Bai R, et al. Oxidative stress: the core pathogenesis and mechanism of alzheimer’s disease. Ageing Res Rev. 2022;77:101619. 35395415 10.1016/j.arr.2022.101619

[13] Markovinovic A, et al. Endoplasmic reticulum-mitochondria signaling in neurons and neurodegenerative diseases. J Cell Sci. 2022;135(3) 10.1242/jcs.248534 35129196

[14] Johri A. Disentangling mitochondria in Alzheimer’s disease. Int J Mol Sci. 2021;22(21):11520. 34768950 10.3390/ijms222111520 PMC8583788

[15] Cheng YC, Huang YC, Huang WL. Heart rate variability in patients with dementia or neurocognitive disorders: A systematic review and meta-analysis. Aust N Z J Psychiatry. 2022;56(1):16–27. 33287558 10.1177/0004867420976853

[16] Nasb M, Tao W, Chen N. Alzheimer’s disease puzzle: delving into pathogenesis hypotheses. Aging Dis. 2024;15(1):43–73. 37450931 10.14336/AD.2023.0608 PMC10796101

[17] Biessels GJ, Despa F. Cognitive decline and dementia in diabetes mellitus: mechanisms and clinical implications. Nat Rev Endocrinol. 2018;14(10):591–604. 30022099 10.1038/s41574-018-0048-7 PMC6397437

[18] Hanyu H. Diabetes-Related dementia. Adv Exp Med Biol. 2019;1128:147–60. 31062329 10.1007/978-981-13-3540-2_8

[19] Liao MT, et al. ACEI and ARB did not reduce the incidence of dementia in patients with atrial fibrillation: A nationwide cohort study. Acta Cardiol Sin. 2013;29(4):323–7. 27122725 PMC4804898

[20] Chen YH, et al. Protective effects of angiotensin receptor blockers on the incidence of dementia in patients with chronic kidney disease: a population-based nationwide study. J Clin Med. 2021;10(21):5175. 34768695 10.3390/jcm10215175 PMC8585022

[21] Nagai M, Hoshide S, Kario K. Hypertension and dementia. Am J Hypertens. 2010;23(2):116–24. 19927134 10.1038/ajh.2009.212

[22] Kuller LH. Statins, lipids and dementia? J Clin Lipidol. 2021;15(1):18–21. 33451927 10.1016/j.jacl.2020.12.011

[23] McGuinness B, et al. Statins for the prevention of dementia. Cochrane Database Syst Rev. 2016;20161:pCd003160. 10.1002/14651858.CD003160.pub3 PMC9346344 26727124

[24] Pendlebury ST. Direct oral anticoagulants and prevention of dementia in nonvalvular atrial fibrillation. Stroke. 2021;52(11):3469–71. 34496623 10.1161/STROKEAHA.121.035664

[25] Williams PS et al. Aspirin for vascular dementia. Cochrane Database Syst Rev, 2000. 2000(4): p. Cd001296. 10.1002/14651858.CD001296 PMC4171457 11034710

[26] Kellar D, Craft S. Brain insulin resistance in alzheimer’s disease and related disorders: mechanisms and therapeutic approaches. Lancet Neurol. 2020;19(9):758–66. 32730766 10.1016/S1474-4422(20)30231-3 PMC9661919

[27] Cholerton B, Baker LD, Craft S. Insulin, cognition, and dementia. Eur J Pharmacol. 2013;719(1–3):170–9. 24070815 10.1016/j.ejphar.2013.08.008 PMC5405627

[28] Sędzikowska A, Szablewski L. Insulin and insulin resistance in Alzheimer’s disease. Int J Mol Sci. 2021;22(18):9987. 34576151 10.3390/ijms22189987 PMC8472298

[29] Crichton GE, et al. Metabolic syndrome, cognitive performance, and dementia. J Alzheimers Dis. 2012;30(Suppl 2):S77–87. 21971405 10.3233/JAD-2011-111022

[30] Borshchev YY, Uspensky YP, Galagudza MM. Pathogenetic pathways of cognitive dysfunction and dementia in metabolic syndrome. Life Sci. 2019;237:116932. 31606384 10.1016/j.lfs.2019.116932

[31] Folstein MF, Folstein SE, McHugh PR. *Mini-mental state. A practical method for grading the cognitive state of patients for the clinician.* J Psychiatr Res, 1975. 12(3): pp. 189 – 98. 10.1016/0022-3956(75)90026-6 1202204

[32] Oscanoa TJ, et al. Estimation of the glomerular filtration rate in older individuals with serum creatinine-based equations: A systematic comparison between CKD-EPI and BIS1. Arch Gerontol Geriatr. 2018;75:139–45. 29304508 10.1016/j.archger.2017.12.007

[33] Cao Z, et al. Associations of BMI and serum urate with developing dementia: a prospective cohort study. J Clin Endocrinol Metab. 2020;105(12):e4688-98. 10.1210/clinem/dgaa638 32918088

[34] García-Ptacek S, et al. Body mass index in dementia. Eur J Clin Nutr. 2014;68(11):1204–9. 25271014 10.1038/ejcn.2014.199

[35] Qizilbash N, et al. BMI and risk of dementia in two million people over two decades: a retrospective cohort study. Lancet Diabetes Endocrinol. 2015;3(6):431–6. 25866264 10.1016/S2213-8587(15)00033-9

[36] O’Brien JT, Thomas A. Vascular Dement Lancet. 2015;386(10004):1698–706. 10.1016/S0140-6736(15)00463-8 26595643

[37] Goulay R, et al. From stroke to dementia: a comprehensive review exposing tight interactions between stroke and Amyloid-β formation. Transl Stroke Res. 2020;11(4):601–14. 31776837 10.1007/s12975-019-00755-2 PMC7340665

[38] Pinkston JB, Alekseeva N, Toledo GE. Stroke and dementia. Neurol Res. 2009;31(8):824–31. 19723451 10.1179/016164109X12445505689643

[39] Iadecola C. The pathobiology of vascular dementia. Neuron. 2013;80(4):844–66. 24267647 10.1016/j.neuron.2013.10.008 PMC3842016

[40] Walker KA, Power MC, Gottesman RF. Defining the relationship between hypertension, cognitive decline, and dementia: a review. Curr Hypertens Rep. 2017;19(3):24. 28299725 10.1007/s11906-017-0724-3 PMC6164165

[41] Birkenhäger WH, Staessen JA. Progress in cardiovascular diseases: cognitive function in essential hypertension. Prog Cardiovasc Dis. 2006;49(1):1–10. 16867845 10.1016/j.pcad.2006.03.001

[42] Lennon MJ, et al. Midlife hypertension and alzheimer’s disease: A systematic review and Meta-Analysis. J Alzheimers Dis. 2019;71(1):307–16. 31381518 10.3233/JAD-190474

[43] Zhang X, et al. Associations of lipids and lipid-lowering drugs with risk of vascular dementia: a mendelian randomization study. Nutrients. 2022;15(1):69. 36615727 10.3390/nu15010069 PMC9824558

[44] van Oijen M, et al. Atherosclerosis and risk for dementia. Ann Neurol. 2007;61(5):403–10. 17328068 10.1002/ana.21073

[45] Brainin M, et al. Post-stroke cognitive decline: an update and perspectives for clinical research. Eur J Neurol. 2015;22(2):229–38. e13-6. 25492161 10.1111/ene.12626

[46] Rost NS, et al. Post-Stroke cognitive impairment and dementia. Circ Res. 2022;130(8):1252–71. 35420911 10.1161/CIRCRESAHA.122.319951

[47] Hachinski V. The convergence of stroke and dementia. Arq Neuropsiquiatr. 2018;76(12):849–52. 30698209 10.1590/0004-282X20180148

[48] Casserly I, Topol E. Convergence of atherosclerosis and alzheimer’s disease: inflammation, cholesterol, and misfolded proteins. Lancet. 2004;363(9415):1139–46. 15064035 10.1016/S0140-6736(04)15900-X

[49] Zhou J, et al. Association between stroke and alzheimer’s disease: systematic review and meta-analysis. J Alzheimers Dis. 2015;43(2):479–89. 25096624 10.3233/JAD-140666

[50] Doyle KP, et al. B-lymphocyte-mediated delayed cognitive impairment following stroke. J Neurosci. 2015;35(5):2133–45. 25653369 10.1523/JNEUROSCI.4098-14.2015 PMC4315838

[51] Cermakova P, et al. Heart failure and dementia: survival in relation to types of heart failure and different dementia disorders. Eur J Heart Fail. 2015;17(6):612–9. 25581033 10.1002/ejhf.222 PMC4674979

[52] Yang M, et al. Interrelationship between alzheimer’s disease and cardiac dysfunction: the brain-heart continuum? Acta Biochim Biophys Sin (Shanghai). 2020;52(1):1–8. 31897470 10.1093/abbs/gmz115

[53] Nguyen TNM, et al. Long-term low-dose acetylsalicylic use shows protective potential for the development of both vascular dementia and alzheimer’s disease in patients with coronary heart disease but not in other individuals from the general population: results from two large cohort studies. Alzheimers Res Ther. 2022;14(1):75. 35624487 10.1186/s13195-022-01017-4 PMC9145441

[54] Gorenflo MP, et al. Association of aspirin use with reduced risk of developing alzheimer’s disease in elderly ischemic stroke patients: A retrospective cohort study. J Alzheimers Dis. 2023;91(2):697–704. 36502331 10.3233/JAD-220901 PMC11388024

[55] Ryan J, et al. Randomized placebo-controlled trial of the effects of aspirin on dementia and cognitive decline. Neurology. 2020;95(3):e320–31. 32213642 10.1212/WNL.0000000000009277 PMC7455352

[56] Parish S, et al. Effects of aspirin on dementia and cognitive function in diabetic patients: the ASCEND trial. Eur Heart J. 2022;43(21):2010–9. 35393614 10.1093/eurheartj/ehac179 PMC9242621

[57] Hussain S, et al. Calcium channel blocker use reduces incident dementia risk in elderly hypertensive patients: A meta-analysis of prospective studies. Neurosci Lett. 2018;671:120–7. 29452176 10.1016/j.neulet.2018.02.027

[58] Tollefson GD. Short-term effects of the calcium channel blocker nimodipine (Bay-e-9736) in the management of primary degenerative dementia. Biol Psychiatry. 1990;27(10):1133–42. 2187540 10.1016/0006-3223(90)90050-c

[59] Nimmrich V, Eckert A. Calcium channel blockers and dementia. Br J Pharmacol. 2013;169(6):1203–10. 23638877 10.1111/bph.12240 PMC3831702

[60] Anekonda TS, et al. L-type voltage-gated calcium channel Blockade with Isradipine as a therapeutic strategy for alzheimer’s disease. Neurobiol Dis. 2011;41(1):62–70. 20816785 10.1016/j.nbd.2010.08.020 PMC2982927

[61] Tae BS, et al. α-Blocker and risk of dementia in patients with benign prostatic hyperplasia: A nationwide population based study using the National health insurance service database. J Urol. 2019;202(2):362–8. 30840545 10.1097/JU.0000000000000209

[62] Frankel JK, Duan Y, Albertsen PC. Is Tamsulosin linked to dementia in the elderly?? Curr Urol Rep. 2018;19(9):69. 29971698 10.1007/s11934-018-0821-0

[63] Coelho BP, et al. Dual effect of doxazosin: anticancer activity on SH-SY5Y neuroblastoma cells and neuroprotection on an in vitro model of alzheimer’s disease. Neuroscience. 2019;404:314–25. 30771511 10.1016/j.neuroscience.2019.02.005

[64] Fournier A, et al. Prevention of dementia by antihypertensive drugs: how AT1-receptor-blockers and dihydropyridines better prevent dementia in hypertensive patients than Thiazides and ACE-inhibitors. Expert Rev Neurother. 2009;9(9):1413–31. 19769454 10.1586/ern.09.89

[65] Gebhard C, et al. Lipid-lowering therapy and the risk of dementia: lessons learned from two decades of controversy. Eur Heart J. 2023;44(21):1855–7. 36896631 10.1093/eurheartj/ehad103

[66] De Felice FG, Ferreira ST. Inflammation, defective insulin signaling, and mitochondrial dysfunction as common molecular denominators connecting type 2 diabetes to alzheimer disease. Diabetes. 2014;63(7):2262–72. 24931033 10.2337/db13-1954

[67] Ma L, Wang J, Li Y. Insulin resistance and cognitive dysfunction. Clin Chim Acta. 2015;444:18–23. 25661087 10.1016/j.cca.2015.01.027

[68] De Felice FG, Lourenco MV, Ferreira ST. How does brain insulin resistance develop in alzheimer’s disease? Alzheimers Dement. 2014;10(1 Suppl):S26–32. 24529521 10.1016/j.jalz.2013.12.004

[69] Luchsinger JA, et al. Hyperinsulinemia and risk of alzheimer disease. Neurology. 2004;63(7):1187–92. 15477536 10.1212/01.wnl.0000140292.04932.87

[70] Burns JM, et al. Peripheral insulin and brain structure in early alzheimer disease. Neurology. 2007;69(11):1094–104. 17846409 10.1212/01.wnl.0000276952.91704.af

[71] Liperoti R, et al. Association between frailty and ischemic heart disease: a systematic review and meta-analysis. BMC Geriatr. 2021;21(1):357. 34112104 10.1186/s12877-021-02304-9 PMC8193864

[72] Giovannini S, et al. Polypharmacy in home care in europe: Cross-Sectional data from the IBenC study. Drugs Aging. 2018;35(2):145–52. 29411310 10.1007/s40266-018-0521-y

[73] Giovannini S, et al. Use of antidepressant medications among older adults in European long-term care facilities: a cross-sectional analysis from the SHELTER study. BMC Geriatr. 2020;20(1):310. 32854659 10.1186/s12877-020-01730-5 PMC7457305

